# Supplementary material for: Anti-senescent drug screening by deep learning-based morphology senescence scoring
Source: Nat Commun. 2021 Jan 11;12:257. doi: 10.1038/s41467-020-20213-0 (PMC7801636; doi:10.1038/s41467-020-20213-0)
Supplement: Supplementary file 1 — Supplementary Information [file 41467_2020_20213_MOESM1_ESM.pdf]

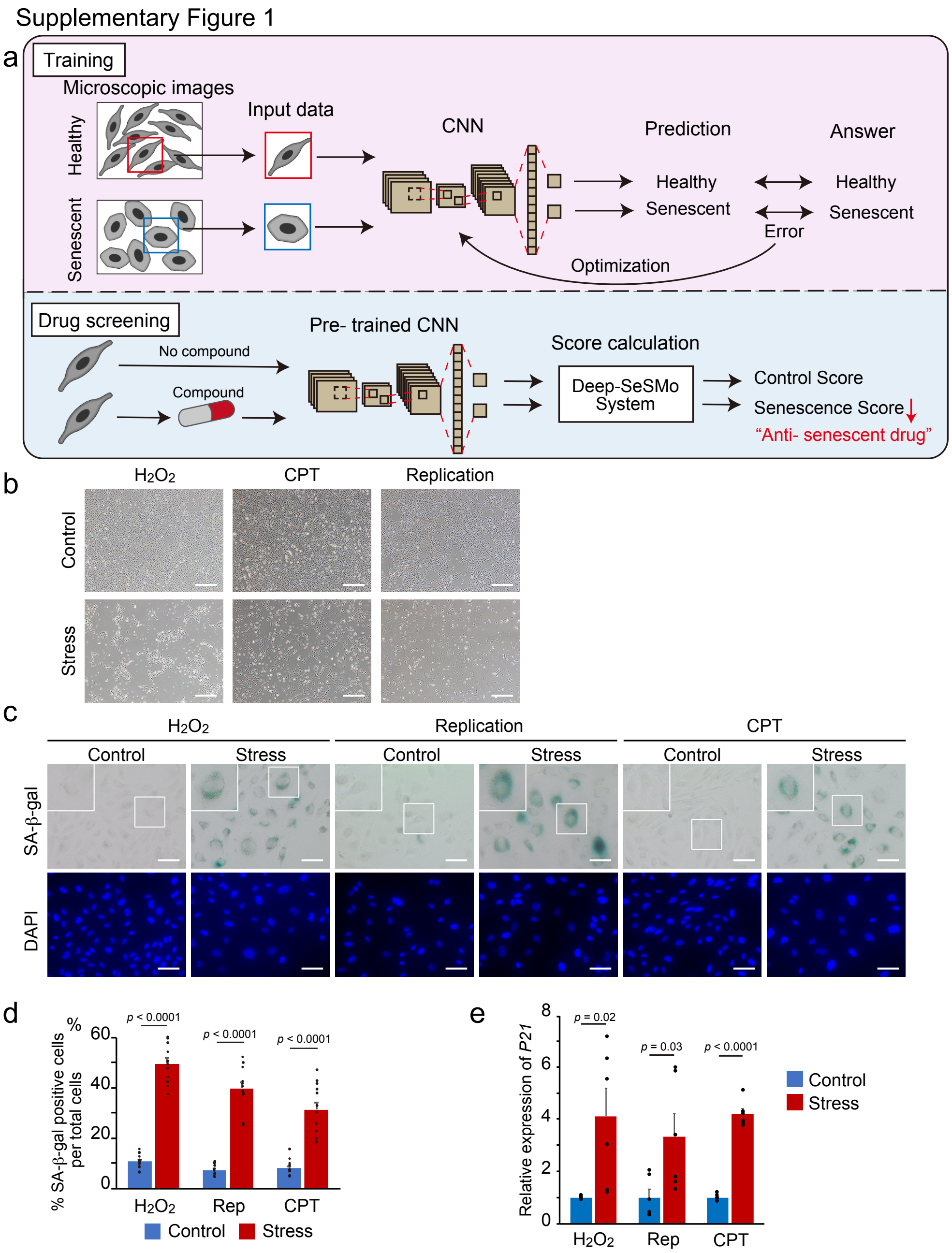

Supplementary Figure 1: Cellular senescence induction in endothelial cells.
a, Concept of the study design. For the CNN training, microscopic images of healthy and senescent cells were acquired; each single cell image was cropped from larger images, and the CNN was trained to identify healthy and senescent cells. For drug screening, we calculated the senescence score of input cells by applying senescence probability output by the last layer of the CNN, named Deep Learning-Based Senescence Scoring System by Morphology (Deep-SeSMo). Senescent cells were incubated with each drug from the library, each senescence score was compared with the control score, and potential anti-senescent drugs were selected. **b,** Phase-contrast images of senescent endothelial cells and control cells. Cellular senescence was induced by three methods: H_2O_2, CPT, and replication. Scale bar, 500 μm. Data are representative of over three independent experiments. **c,** SA-β-gal activity in control and stressed HUVECs. Cellular senescence was induced by three methods: H_2O_2 (four days), CPT (two days), and replication (10 passages). Scale bar; 100 μm. Data are representative of two independent experiments. **d,** Percentage of SA-β-gal positive cells per total cells (n = 12 images over two independent experiments). **e,** qRT-PCR analysis determined the relative expression of *P21* in control and senescent cells (n = 6 biological replicates). CNN: convolutional neural network, CPT: camptothecin, Rep: Replication. Data are shown as mean ± s.e.m. p-values by two-sided student's t-test.

Supplementary Figure 2

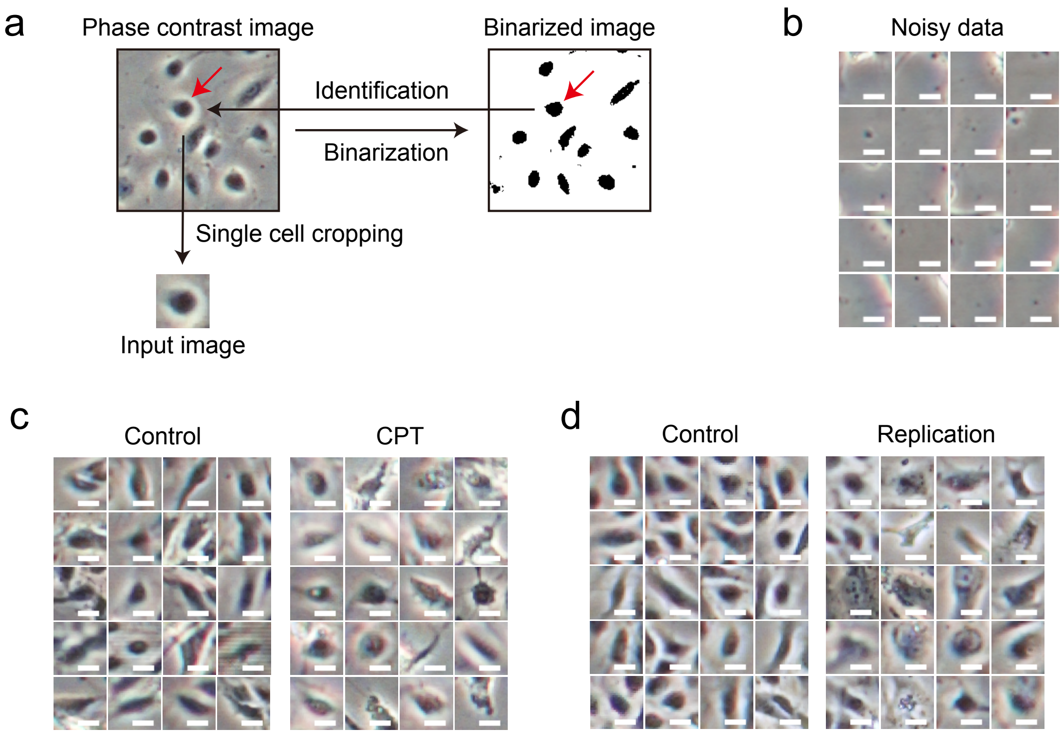

e

	Layer	Parameter
1	Convolution	32, (3, 3)
2	Convolution	32, (3, 3)
3	MaxPooling	2, 2
4	Dropout	0.5
5	Convolution	64, (3, 3)
6	Convolution	128, (3, 3)
7	MaxPooling	2, 2
8	Dropout	0.5
9	Flatten	
10	Dense	256
11	Dropout	0.5
12	Dense	2

Supplementary Figure 2: Input data preparation.
a, Schema for input image preparation. Phase contrast images were binarized, and the centers of the nuclei were identified. Input images were cropped around the nuclei with an area of 50 × 50 pixels. **b,** Representative images of excluded noisy patches, detected as small particles. Data are representative of over three independent experiments. **c, d,** Representative input images of (**c**) CPT- and (**d**) replication-induced senescent endothelial cells were cropped from phase-contrast microscopic images using the OpenCV-based script at single cell resolution. Data are representative of over three independent experiments. **e,** Construction of the CNN. It consists of four convolutional layers, two max-pooling layers, and two fully connected layers. Scale bar, 7.1 µm. CPT: camptothecin.

Supplementary Figure 3

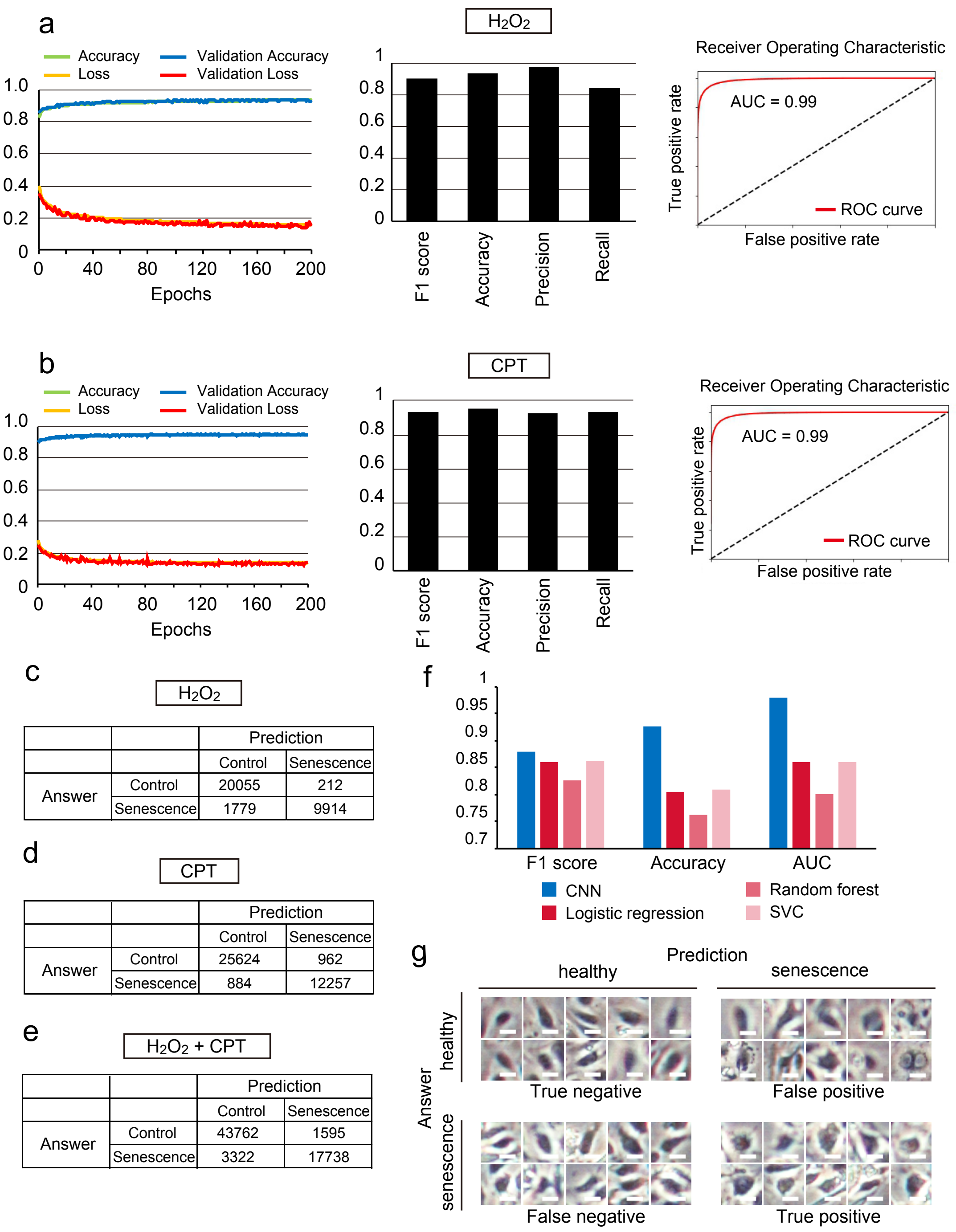

c — H_2O_2

		Prediction	
		Control	Senescence
Answer	Control	20055	212
	Senescence	1779	9914

d — CPT

		Prediction	
		Control	Senescence
Answer	Control	25624	962
	Senescence	884	12257

e — H_2O_2 + CPT

		Prediction	
		Control	Senescence
Answer	Control	43762	1595
	Senescence	3322	17738

Supplementary Figure 3: CNN training.

a, Learning curve (left graph), several indexes for evaluation (middle graph), and AUC of the ROC curve (right graph) show the performance of the CNN trained by H_2O_2-induced cellular senescence data. **b,** Learning curve (left graph), several indexes for evaluation (middle graph), and AUC of the ROC curve (right graph) demonstrate the performance of the CNN trained by CPT-induced cellular senescence data. **c, d, e,** Confusion matrix shows the true positive, true negative, false positive, and false negative prediction and answer in the training of (**c**) H_2O_2-, (**d**) CPT-, and (**e**) both H_2O_2- and CPT-induced senescent HUVECs. **f,** The performance of machine learning; Logistic regression, Random forest, Support vector classifier (SVC), and CNN. **g,** Representative images of true positive, true negative, false positive, and false negative predictions by CNN. Scale bar, 7.1 μm. Data are representative of over three independent experiments. CPT: camptothecin, CNN: convolutional neural network, SVC: Support vector classifier.

Supplementary Figure 4

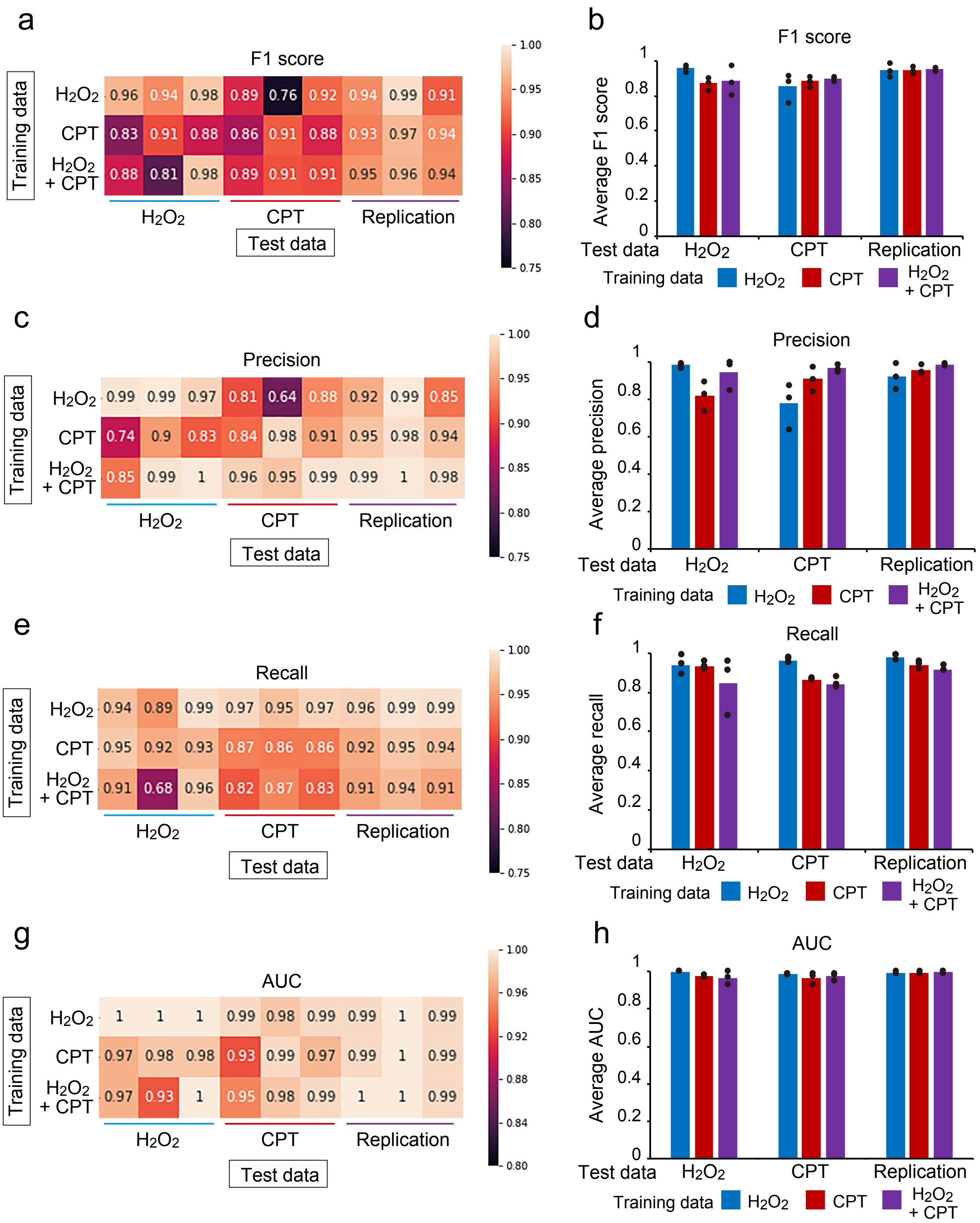

Supplementary Figure 4: CNN generalizability.

a, The heatmap shows the F1 score of CNN prediction in each test dataset. Three independent experiments and evaluations were conducted for each senescence induction method. **b,** Macro-averaged F1 score for each evaluation. **c,** Precision of each set of test data. **d,** Macro-averaged precision in each evaluation. **e,** Recall in each set of test data. **f,** Macro-averaged recall in each evaluation. **g,** AUC of the ROC curve for each set of test data. **h,** Macro-averaged AUC for each evaluation. **b,d,f,h,** (n = 3 independent experiments). CPT: camptothecin.

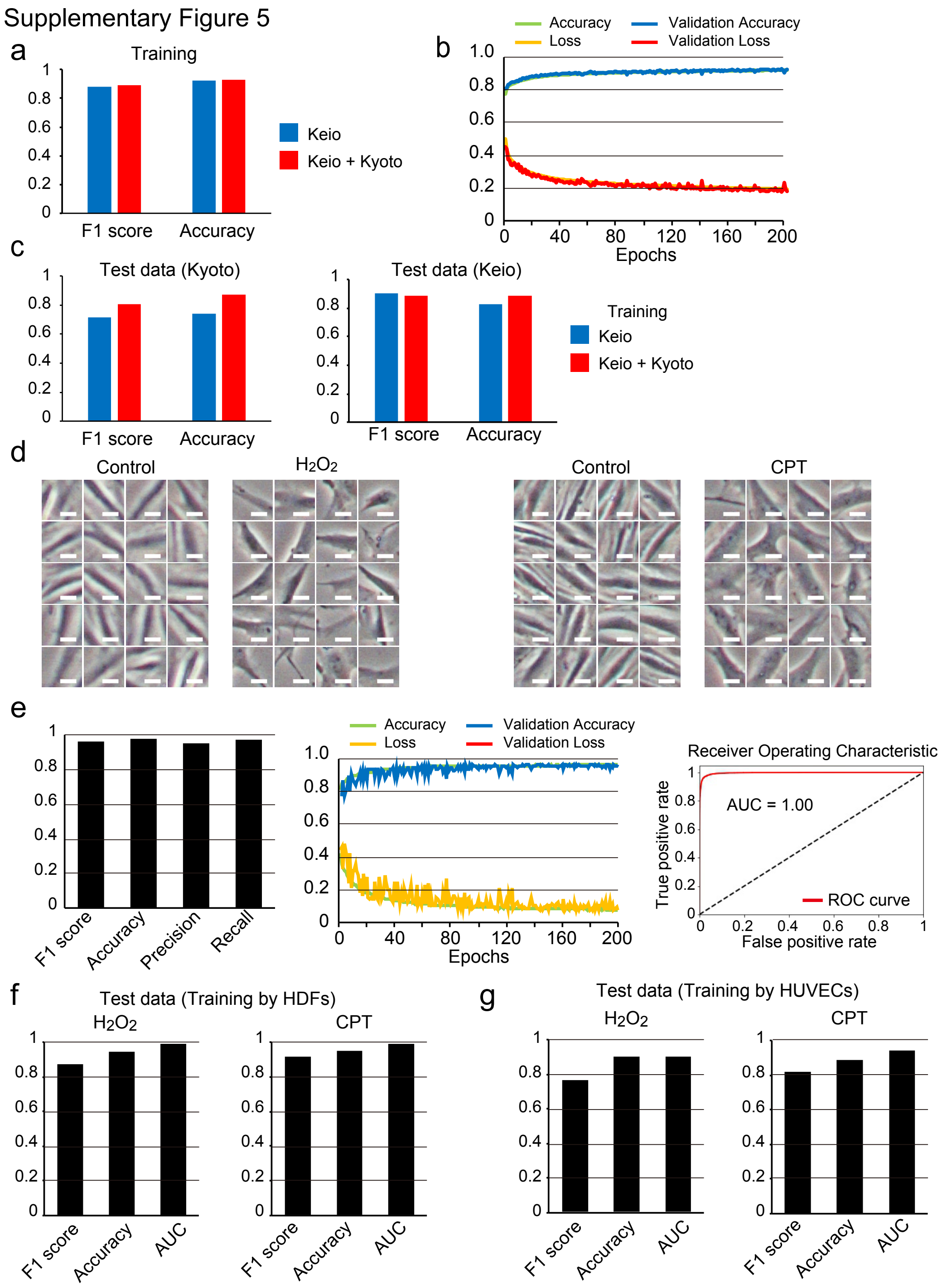

Supplementary Figure 5: CNN performance at another institute and in another cell type.

a, Performance of CNN trained by both Keio and Kyoto datasets, or Keio datasets alone. **b,** Learning curve through the CNN training in the both Keio and Kyoto datasets. Accuracy and loss in the training data, and validation accuracy and validation loss in the validation data show the process of training. **c,** Comparison of performance using the Kyoto test data (left graph) or Keio test data (right graph), between CNN trained by both Keio and Kyoto datasets, and CNN trained by Keio datasets alone. **d,** Representative input images of H_2O_2 (left panel) and CPT (right panel)-induced senescent HDFs at the single cell resolution. Scale bar, 7.1 µm. Data are representative of three independent experiments. **e,** Several indexes (left graph), learning curve (middle graph), and AUC of the ROC curve (right graph) show the performance of the CNN trained on the healthy and senescent HDFs. **f,** The performance of HDF-trained CNN in test datasets using H_2O_2 (left graph) or CPT (right graph)-induced senescent HDFs. **g,** The performance of HUVEC-trained CNN in test datasets using H_2O_2 (left graph) or CPT (right graph)-induced senescent HDFs. CPT: camptothecin.

Supplementary Figure 6

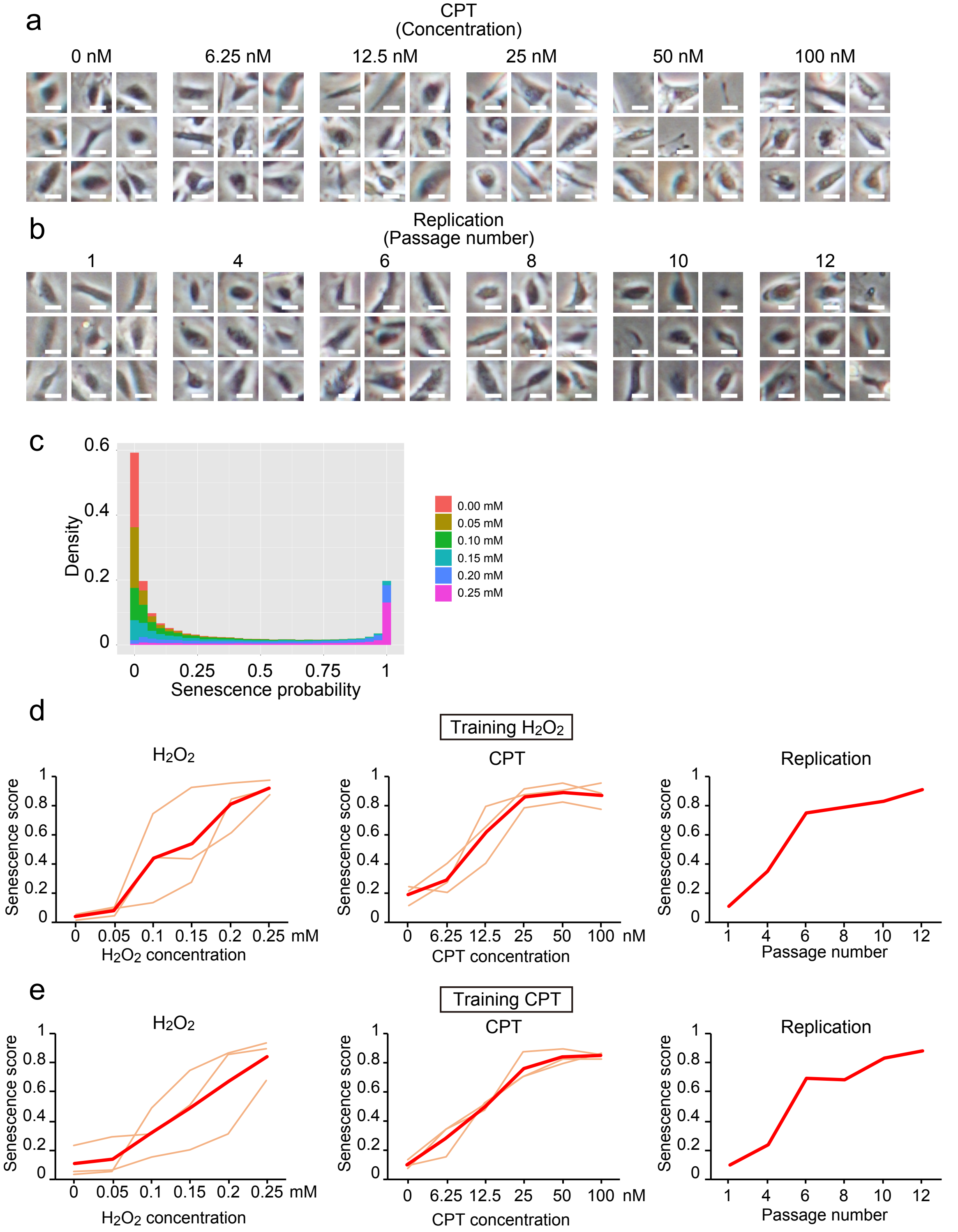

Supplementary Figure 6: Development of Deep-SeSMo.

a, b, Representative input images of HUVECs with (**a**) various concentrations of CPT and (**b**) several passage numbers. Scale bar, 7.1 µm. Data are representative of three independent experiments. **c,** Density histogram shows the senescence probability for each HUVEC with several concentrations of H_2O_2. Data are representative of three independent experiments. **d, e,** Senescence score calculated by Deep-SeSMo using CNNs trained by (**d**) H_2O_2- and (**e**) CPT-induced senescent HUVECs with various concentrations of H_2O_2 and CPT and passage numbers. The thin line and thick line demonstrate each score and the average score, respectively. CPT: camptothecin.

Supplementary Figure 7

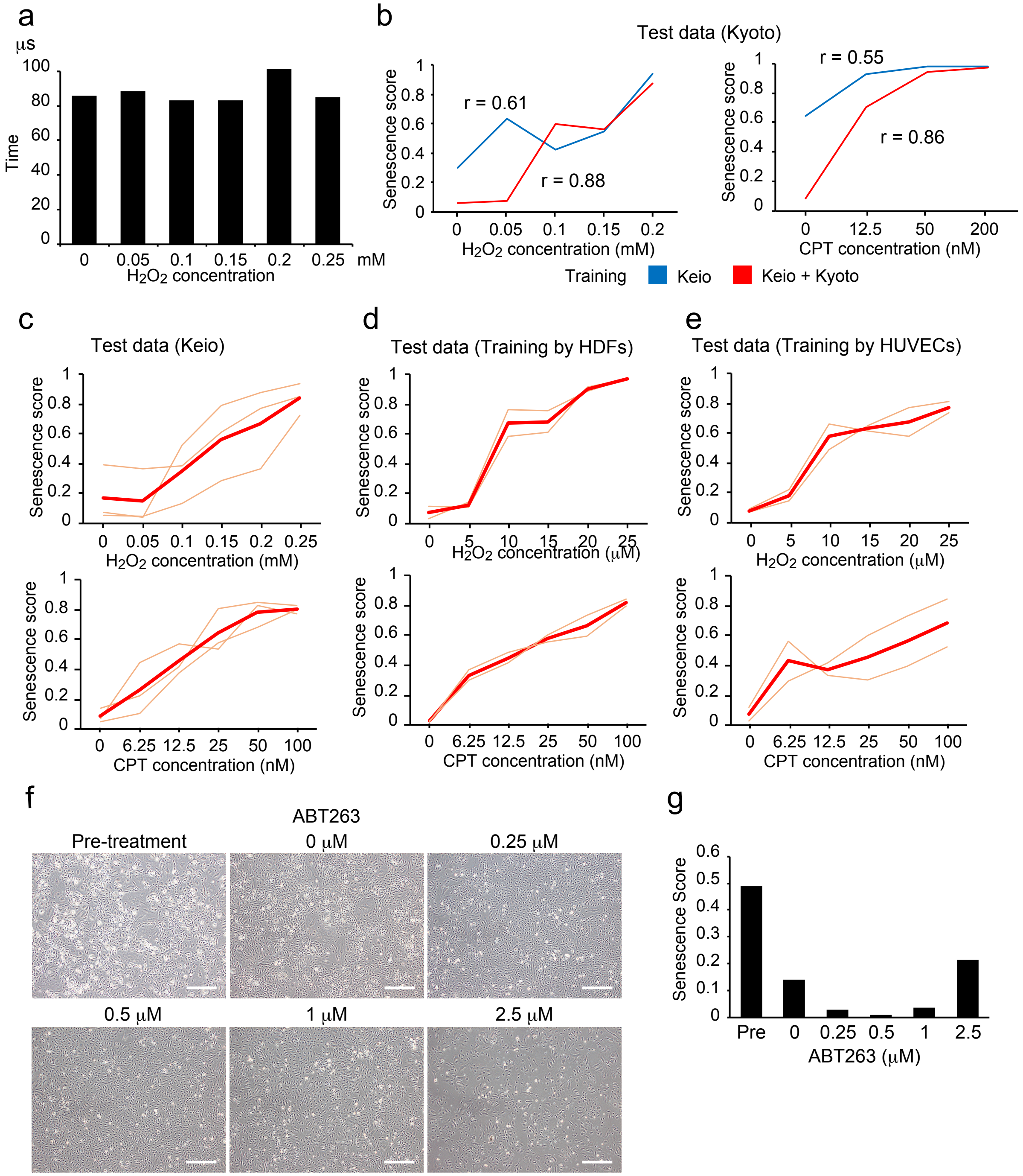

Supplementary Figure 7: Generalizability of senescence score

a, Time to calculate the senescence score by Deep-SeSMo from one phase contrast image. Data are representative of over three independent experiments. **b,** Senescence score calculated by Deep-SeSMo in the test datasets obtained at Kyoto University, using CNNs trained on both the Keio and Kyoto datasets, or trained on the Keio datasets alone. **c,** Senescence score in the test datasets obtained at Keio University using CNNs trained on the both Keio and Kyoto datasets. The thin line and thick line demonstrate each score and the average score, respectively. **d, e,** Senescence score in the test datasets of HDFs evaluated by (d) CNNs trained on the HDFs or (e) HUVECs. The thin line and thick line demonstrate each score and the average score, respectively. **f,** Representative images of HUVECs, which were a mixture of young and old cells, treated with several concentrations of ABT263 for 72 hours. Scale bar, 500 μm. Data are representative of two independent experiments. **g,** Senescence score calculated by Deep-SeSMo showed the senolytic effect of ABT263. Data are representative of two independent experiments. CPT: camptothecin.

Supplementary Figure 8

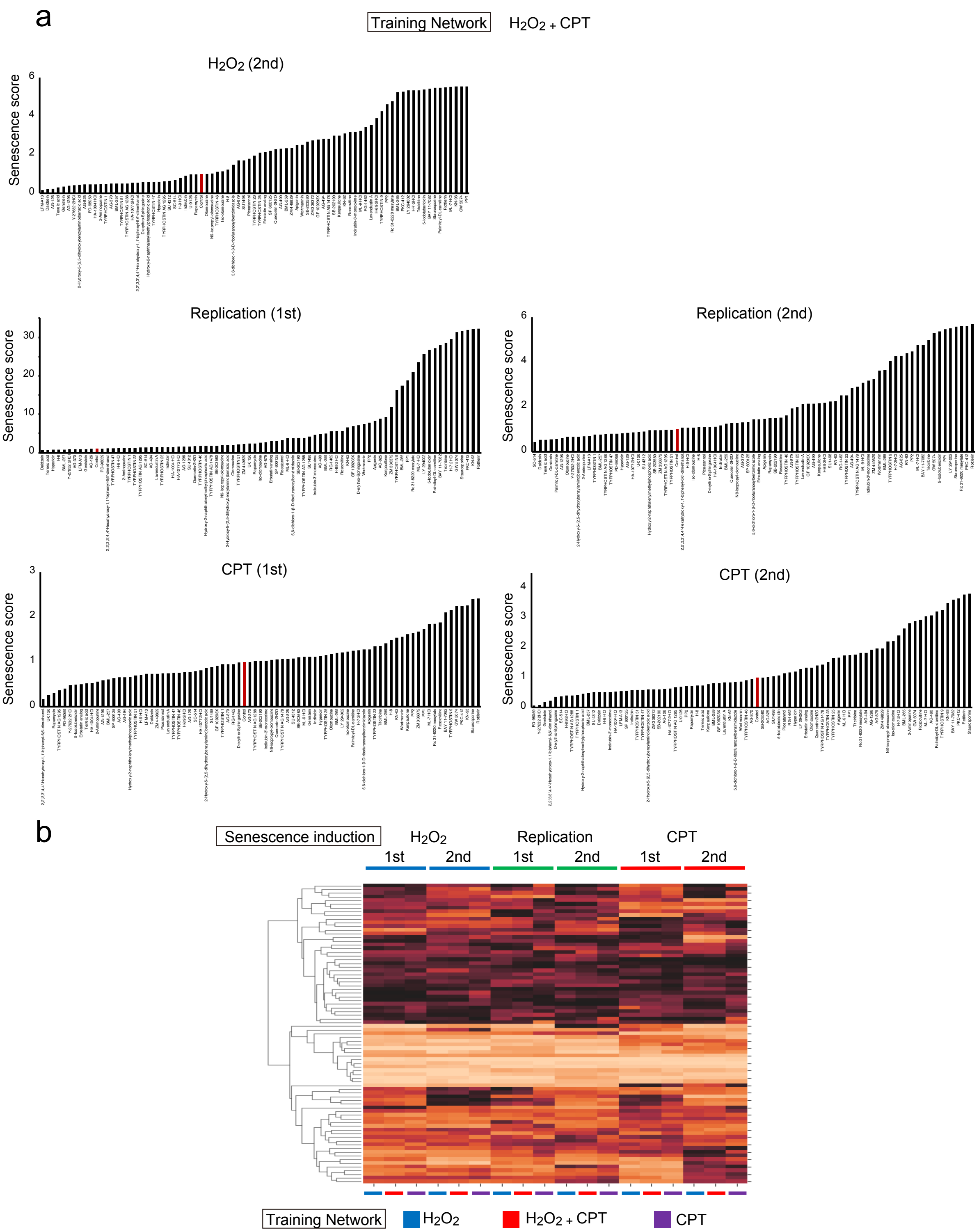

Supplementary Figure 8: Drug screening using Deep-SeSMo

a, b, Eighty compounds were added to HUVECs, and cellular senescence was induced by three methods: H_2O_2, CPT, and replication. The induction was repeated twice for each condition. The senescence score was calculated by Deep-SeSMo. **a,** Graph shows the normalized senescence score using Deep-SeSMo trained by both H_2O_2 and CPT for each compound normalized by a control score. **b,** Heatmap demonstrates the senescence score ranking for each experiment. CPT: Camptothecin. CNN: Convolutional neural network. Deep-SeSMo: Deep Learning-Based Senescence Scoring System by Morphology. HUVECs: Human umbilical vein endothelial cells. CPT: camptothecin.

Supplementary Figure 9

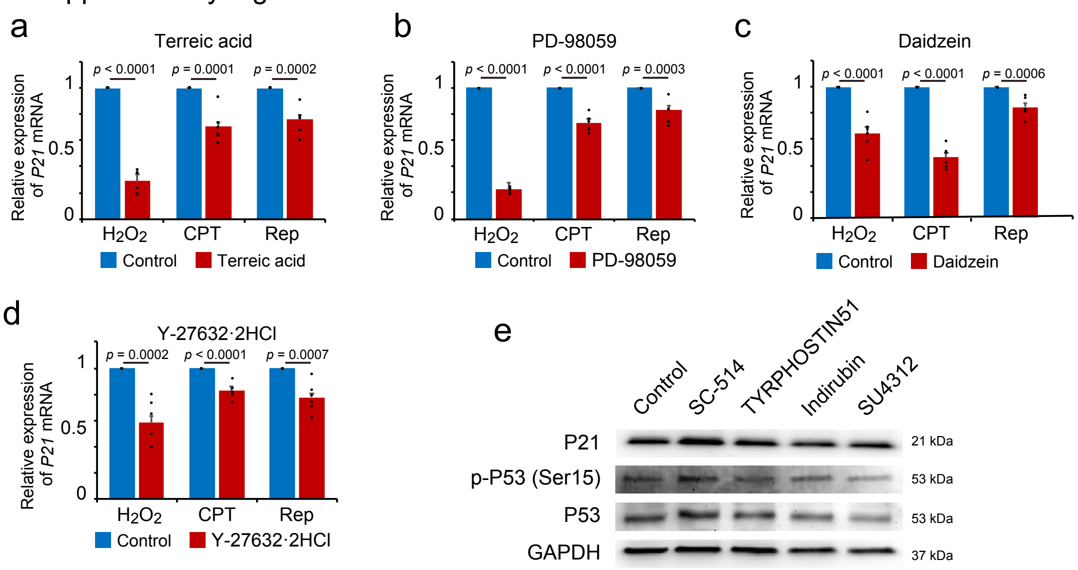

f

Top10 genes (*p*-value)

Gene name	*p*-value	Fold change
MTHFSD	6.46E-05	1.58972814
ASCC1	0.000236395	1.931667741
TMEM184B	0.000250038	1.92514839
SIGIRR	0.000603025	2.390774597
BBC3	0.000776761	1.406509564
RCHY1	0.000867038	0.375769013
TBL1XR1	0.000888327	0.644882808
CTU1	0.000898887	1.190924385
AC106795.1	0.000925125	1.653767043
ZNF787	0.000961321	1.3727699

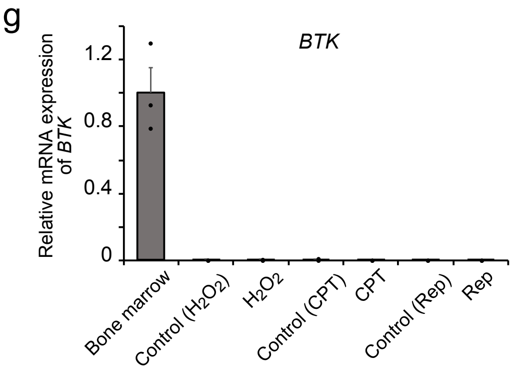

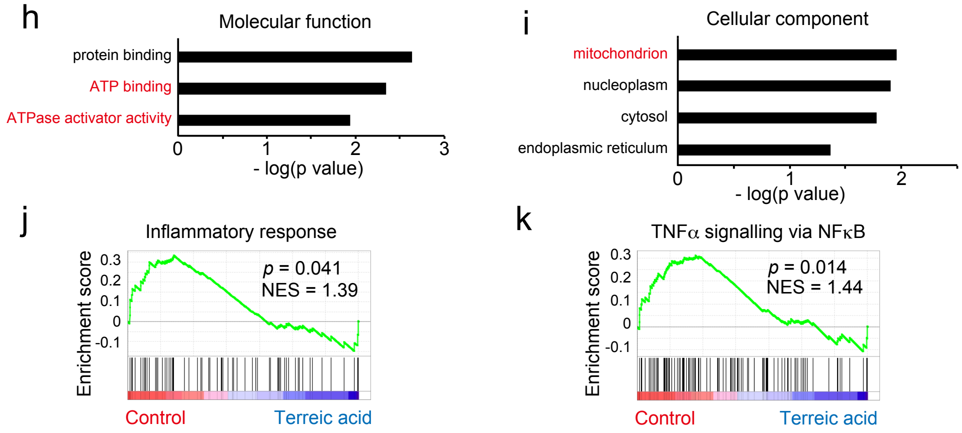

Supplementar Figure 9: Anti- senescent and anti- inflammatory effects of the four compounds

a-d, qRT-PCR analysis determined *P21* mRNA expression in H_2O_2-, CPT-, or replication-induced senescent HUVECs treated with the top four compounds (n = 6 biological replicates). **e,** Western blotting of P21, P53, and Ser15 phosphorylation of P53. The four drugs, which produced almost the same senescent score as the control by Deep-SeSMo, were added to HUVECs with H_2O_2. GAPDH was used as an internal control. Data are representative of two independent experiments. **f,** The top 10 genes selected by differential gene expression analysis among the top four compounds and control. Genes were sorted by *p*-value. **g,** Relative expression of *BTK* mRNA detected by qRT-PCR (n = 3 biological replicates). **h, i,** GO analysis, categorized as (**h**) Cellular component, and (**i**) Molecular function, of genes upregulated in HUVECs treated with terreic acid compared with control. **j, k,** GSEA of genes associated with (**j**) inflammatory response, and (**k**) TNFα signalling. Data are shown as mean ± s.e.m. *p*-values by two-sided student's t-test. CPT: camptothecin, Rep: Replication, NES: normalised enrichment score.
