# Supplementary material for: Anti-senescent drug screening by deep learning-based morphology senescence scoring
Source: Nat Commun. 2021 Jan 11;12:257. doi: 10.1038/s41467-020-20213-0 (PMC7801636; doi:10.1038/s41467-020-20213-0)
Supplement: Supplementary file 3 — Reporting Summary [file 41467_2020_20213_MOESM3_ESM.pdf]

nature research

Corresponding author(s): Shinsuke Yuasa

Last updated by author(s): Nov 11, 2020

Reporting Summary

Nature Research wishes to improve the reproducibility of the work that we publish. This form provides structure for consistency and transparency in reporting. For further information on Nature Research policies, see our Editorial Policies and the Editorial Policy Checklist.

Statistics

For all statistical analyses, confirm that the following items are present in the figure legend, table legend, main text, or Methods section.

n/a	Confirmed	
☐	☒	The exact sample size (n) for each experimental group/condition, given as a discrete number and unit of measurement
☐	☒	A statement on whether measurements were taken from distinct samples or whether the same sample was measured repeatedly
☐	☒	The statistical test(s) used AND whether they are one- or two-sided<br>*Only common tests should be described solely by name; describe more complex techniques in the Methods section.*
☒	☐	A description of all covariates tested
☒	☐	A description of any assumptions or corrections, such as tests of normality and adjustment for multiple comparisons
☐	☒	A full description of the statistical parameters including central tendency (e.g. means) or other basic estimates (e.g. regression coefficient) AND variation (e.g. standard deviation) or associated estimates of uncertainty (e.g. confidence intervals)
☐	☒	For null hypothesis testing, the test statistic (e.g. F, t, r) with confidence intervals, effect sizes, degrees of freedom and P value noted<br>*Give P values as exact values whenever suitable.*
☒	☐	For Bayesian analysis, information on the choice of priors and Markov chain Monte Carlo settings
☒	☐	For hierarchical and complex designs, identification of the appropriate level for tests and full reporting of outcomes
☐	☒	Estimates of effect sizes (e.g. Cohen's d, Pearson's r), indicating how they were calculated

Our web collection on statistics for biologists contains articles on many of the points above.

Software and code

Policy information about availability of computer code

Data collection	We used fastqc v0.11.7, Trimmomatic v0.36, , cufflinks v2.2.1, hisat2 v2.5.0, stringtie v1.3.3, samtools v1.3.2 for RNA sequence data. Custom codes can be obtained at [https://github.com/Dai-Kusumoto/Deep-SeSMo]
Data analysis	We used matplotlib v2.0.2, Keras v2.1.2, numpy v1.12.1, opencv-python v3.3.0.10, pandas v0.20.1, Pillow v4.1.1, scikit-learn v0.19.0, seaborn v0.7.1, tensorflow-gpu v1.4.0 and tqdm v4.29.0 (Python), and dplyr v1.0.0, ballgown v2.4.2 and gglot2 v3.3.2 (R) for data analysis.<br>Custom codes can be obtained at [https://github.com/Dai-Kusumoto/Deep-SeSMo]

For manuscripts utilizing custom algorithms or software that are central to the research but not yet described in published literature, software must be made available to editors and reviewers. We strongly encourage code deposition in a community repository (e.g. GitHub). See the Nature Research guidelines for submitting code & software for further information.

Data

Policy information about availability of data

All manuscripts must include a data availability statement. This statement should provide the following information, where applicable:

- Accession codes, unique identifiers, or web links for publicly available datasets
- A list of figures that have associated raw data
- A description of any restrictions on data availability

Source data for figures are provided with the paper. For RNA sequence data in Figure 4 and Supplementary Figure 9, raw data have been deposited in DDBJ Sequence Read Archive (DRA) with the accession code “DRA010959 [http://trace.ddbj.nig.ac.jp/DRASearch/submission?acc=DRA010959]”.

Field-specific reporting

Please select the one below that is the best fit for your research. If you are not sure, read the appropriate sections before making your selection.

☒ Life sciences ☐ Behavioural & social sciences ☐ Ecological, evolutionary & environmental sciences

For a reference copy of the document with all sections, see nature.com/documents/nr-reporting-summary-flat.pdf

Life sciences study design

All studies must disclose on these points even when the disclosure is negative.

Sample size	The number of input dataset is highly important for successful CNN training. Previously, we reported that over 32,000 blocks were required for classification of induced pluripotent stem cells-derived endothelial cells (Kusumoto et. al. Stem Cell Reports. 2018). Therefore, we determined that over 32,000 input datasets are necessary for this study. Moreover, larger datasets are better for CNN training and we therefore used over 100,000 input datasets for this study.
Data exclusions	We excluded the analysis obtained from cells which showed obviously bad condition, because they would give wrong labels.
Replication	Data are representative of over three (Figures 1a, 1i, 2a, 2e, 2f, and Supplementary figures 1b, 2b, 2c, 2d, 3g, 5d, 6a, 6b, 6c, 7a,) or two (Figures 3a, 3c, 3d, 3e, 4a, 4c Supplementary figures 1c, 7f, 7g, 9e.) independent experiments. All attempts at replication were successful.
Randomization	All microscopic images for analysis were randomly acquired in each condition.
Blinding	Blinding was not applied to almost experiments, because data collection and analysis was performed by same investigators, and blinding was technically difficult. However, for the key analysis, we performed blinding analysis. For development of Deep-SeSMo, one evaluation of senescence score was also performed by blinding. Drug screening for anti-senescense was performed by blinding until all data were collected and analyzed.

Reporting for specific materials, systems and methods

We require information from authors about some types of materials, experimental systems and methods used in many studies. Here, indicate whether each material, system or method listed is relevant to your study. If you are not sure if a list item applies to your research, read the appropriate section before selecting a response.

Materials & experimental systems

n/a	Involved in the study	
☐	☒	Antibodies
☐	☒	Eukaryotic cell lines
☒	☐	Palaeontology and archaeology
☒	☐	Animals and other organisms
☒	☐	Human research participants
☒	☐	Clinical data
☒	☐	Dual use research of concern

Methods

n/a	Involved in the study	
☒	☐	ChIP-seq
☒	☐	Flow cytometry
☒	☐	MRI-based neuroimaging

Antibodies

Antibodies used	The following primary antibodies were used in the study.<br><br>P21 (#2947, 12D1, Cell Signaling)<br>P53 (#ab1101, DO-1)<br>phospho-P53 (#9284, Cell Signaling)<br>P16INK4a (#ab108349, Abcam)<br>GAPDH (#2118, 14C10, Cell Signaling)
Validation	The following antibodies have been validated by their manufacturers and are widely used in the scientific community.<br><br>P21 (#2947, 12D1, Cell Signaling, https://www.cellsignal.com/products/primary-antibodies/p21-waf1-cip1-12d1-rabbit-mab/2947?Ntk=Products&Ntt=2947),<br>P53 (#ab1101, DO-1, Abcam, https://www.abcam.co.jp/p53-antibody-do-1-chip-grade-ab1101.html)<br>phospho-P53 (#9284, Cell Signaling, https://en.cellsignal.jp/products/primary-antibodies/phospho-p53-ser15-antibody/9284?Ntk=Products&Ntt=9284),<br>P16INK4a (#ab108349, Abcam, https://www.abcam.co.jp/cdkn2ap16ink4a-antibody-epr1473-c-terminal-ab108349.html)<br>GAPDH (#2118, 14C10, Cell Signaling, https://www.cellsignal.jp/products/primary-antibodies/gapdh-14c10-rabbit-mab/2118?

Ntk=Products&Ntt=2118)

Eukaryotic cell lines

Policy information about cell lines

Cell line source(s)	Human umbilical vein endothelial cells (HUVEC) was purchased from KURABO (#KE-4109) . TIG-114 (Human diploid fibroblast) was purchased from Japanese Collection of Research Bioresources (JCRB) Cell Bank.
Authentication	None of the cell lines used were authenticated
Mycoplasma contamination	The cell lines were not tested for Mycoplasma contamination.
Commonly misidentified lines (See ICLAC register)	We did not use commonly misidentified lines.
